# Estradiol-17ß, progesterone, and oviductal changes in muscovy ducks *(Cairina moschata forma domestica)* during reproductive phases

**DOI:** 10.1186/s12917-025-04737-5

**Published:** 2025-04-14

**Authors:** Martin Linde, Axel Wehrend, Abbas Farshad

**Affiliations:** Veterinary Clinic for Reproductive Medicine and Neonatology, Justus-Liebig-University of Giessen, 35392 Giessen, Germany

**Keywords:** Reproduction cycle, Oviduct, Estradiol-17ß, Progesterone, Muscovy ducks

## Abstract

**Background:**

The role of Muscovy ducks in industrial poultry production is gaining importance; however, their reproductive physiology remains poorly understood. This study examines changes in reproductive performance in female Muscovy ducks *(Cairina moschata forma domestica)* by analyzing estradiol-17β and progesterone levels along with detailed anatomical examinations of the oviduct. Thirty-five young female ducks emerged together, each hatched after a synchronized incubation period of thirty-five days, were randomly divided into groups and housed in outdoor aviaries. Each aviary was equipped with a feeding trough, a water container, and a nesting box. The ducks were artificially hatched under controlled conditions. At the outset of the experiment, the ducks exhibited no laying activity. Within the investigation, the serum estradiol-17β and progesterone levels, along with the macroscopic and microscopic structure of the oviduct, were evaluated.

**Results:**

Ducks were euthanized at various intervals after laying their 1st, 2nd, 4th, 6th, 10th, 15th, and 20th eggs. Analyses focused on endocrine status and the anatomical structure of the oviducts. Results indicate that the serum concentrations of Estradiol-17ß and Progesterone progressively increase as the transition into the laying period occurs, reaching their peak during the laying phase. However, upon transitioning to the brooding period, hormone levels in the blood serum notably decrease. Oviduct length varied significantly throughout the reproductive cycle, with a mean of 107 ± 9 mm (SD) in juveniles. It peaked at a mean of 632 ± 58 mm (SD) after the tenth egg and then declined to a mean of 235 ± 87 mm (SD) by the 35th day of brooding.

**Conclusion:**

This study provides detailed information on the reproductive physiology of Muscovy ducks, highlighting the significant hormonal and anatomical changes that occur throughout their reproductive cycle.

## Background

Prompt and accurate detection of mating readiness and artificial insemination is crucial for successful outcomes. Hence, a thorough clinical assessment of both the external and internal female genitalia is crucial [1, 2]. Numerous studies have explored the reproductive cycles of poultry species, resulting in a wealth of knowledge regarding the physiological and anatomical changes that occur in the female genital tract during sexual maturation and the reproductive cycle [3–6]. These studies provide valuable insights into the physiological, macroscopic [3, 4], and microscopic [3] alterations in the female genital tract during sexual maturation and the reproductive cycle in poultry species.

The oviduct undergoes morphological changes during the reproductive cycle [4, 7]. In chickens, it appears as a thin tube before laying [8], expanding to approximately 70 cm during laying. Outside the laying period, it shrinks back to a thin tube. These changes are caused by hyperplastic and hypertrophic processes [7]. In addition, the weight and length of the oviduct are correlated with the reproductive cycle stage [9]. In chickens, the oviduct mass increases from 0.02 g to 60 g during laying and then decreases to 3–5 g during rest. Cell mass and number also change significantly [7]. The magnum grows from 19 to 49% of the oviduct during laying and then reduces to 25% during rest. The shell gland and vagina also change in size [7]. In wild ducks, the oviduct undergoes similar transformations, with the magnum increasing by 280% [3]. Moreover, the other sections of the oviduct also exhibit a significant increase in length and width, with the infundibulum increasing by 78%, the isthmus by 85%, the uterus by 66%, and the vagina by 25% [4, 10]. Additionally, chicken’s reproductive tract undergoes significant histomorphological changes during the reproductive cycle, and tubular mucosal gland cells undergo the formation, secretion, and involution stages [11]. In the juvenile oviducts, the tunica mucosa consists mostly of stromal cells. As the oviduct matures, tubular glands develop and increase in size until the peak laying period and then regress during rest [11, 12] whereas increased lymphocyte and plasma cell infiltration occurs during laying [13]. In wild ducks, the tunica muscularis and mucosa also undergo changes; the thickness of the magnum muscularis decreases during development and increases at rest. The lamina propria mucosae and height of mucosal folds increase toward the laying stage, with tubular glands filling the lamina propria and increasing in diameter by 58.6% [3]. Sperm reservoir tubules develop during sexual maturation and increase in diameter until peak laying [10]. After laying, the tubular glands in the magnum, isthmus, and shell gland reduce in size and degenerate, with increased connective tissue proliferation and cell debris in the gland lumina [14].

Conversely, despite the increasing economic importance of Muscovy ducks, studies on their reproductive physiology compared with other poultry species are lacking, which has a significant effect on global production [15, 16]. Hormone levels are closely associated with the morphological changes in the female reproductive tract throughout the reproductive cycle in domestic poultry species [17]. Although hormonal manipulation of reproduction in poultry is not currently widespread, the increasing demand for poultry meat may make it necessary [15, 16]. Thus, the primary goal of this study was to examine potential changes in the reproductive efficiency of female Muscovy ducks *(Cairina moschata forma domestica)* within the Anatidae family [18, 19]. Additionally, this study included a detailed analysis of the levels of estradiol-17β and progesterone, along with a comprehensive evaluation of anatomical characteristics of the oviduct.

## Methods

### Experimental design and procedures

The research involved 35 juvenile female Muscovy ducks *(Cairina moschata forma domestica)* from a specific breeding line and family to ensure uniformity in genetic background. This selection was made to reduce genetic variability and provide more consistent results, based on recommendations from Sosa-Madrid et al. [20]. The analysis took place at the Clinic for Reproductive Medicine and Neonatology at Justus-Liebig University of Giessen, focusing on hormonal and morphological changes throughout their reproductive cycle. The ducks exhibited no egg-laying behavior at the start of investigation. Incubation conditions were standardized, with specified parameters for duration (1–35 days), temperature (36.8–37.3 °C), humidity (75–93%), and egg turning frequency (0–6 times per day). Offspring were raised separately by sex. Clinical assessments confirmed the animals were healthy. Nesting boxes were checked daily from 8 to 9 a.m. for egg presence. If an egg was found, individuals in the aviary were digitally screened for egg-laying activities. Subsequently, at least three ducks were euthanized in each phase. For example, three “infantile” ducks, which were fully feathered but not yet fully grown or sexually mature, were euthanized at 70 days of age. Three “juvenile” ducks, which were fully grown and ready to mate but had not yet laid eggs, were euthanized between 275 and 289 days of age. To track changes during egg laying, three ducks per group were euthanized 12 h after laying their first, second, fourth, sixth, tenth, and twentieth eggs, and four ducks after laying their fifteenth egg, between 327 and 344 days of age. Additionally, four ducks were euthanized five days after the start of incubation, at 372 to 384 days of age. Finally, three ducks were euthanized 35 days after the completion of incubation, at 384, 387, and 389 days of age.

### Determining the endocrine status of estradiol-17ß and progesterone

Blood samples were collected using serum blood tubes (Sarstedt, Monovette, 10 ml) during euthanasia. To minimize daily hormone fluctuations, all procedures were consistently performed between 6:00 and 7:00 pm. After collection, the samples were centrifuged at 3500 rpm for 20 min. The resulting serum was then transferred to sample tubes (Gosselin, 8 ml) and stored at − 20 °C for up to one week after each slaughter until further analysis. The levels of estradiol-17ß and progesterone in the blood sera were determined using a well-established laboratory technique known as radioimmunoassay test [21]. For estradiol-17β determination, the intra-assay coefficient of variation ranged from 6.0 to 11.4%, and the inter-assay coefficient of variation ranged from 13.1 to 13.2%. The lower detection limit was 0.4 pg/ml (1.47 pmol/ml), whereas for progesterone determination, the intra-assay coefficient of variation ranged from 8.8 to 9.6%, and the inter-assay coefficient of variation ranged from 8.9 to 11.3%. The lower limit was 0.1 ng/ml (0.318 nmol/ml).

### Oviduct anatomical examination

For the macroscopic examination of the oviduct, 35 ducks were weighed and precisely documented at the time of euthanasia. Following removal, the oviduct was fully extended and measured from its entry point in the cloaca to the end of the infundibular tube. The diameter was measured at 0.5 cm intervals, and the average diameter was calculated from these measurements. Additionally, the largest yolk sphere, defined as the yolk with the greatest diameter among those present in the in-situ ovary, was measured using a caliper (ATORN; INOX 150 mm, linear scale, accuracy 0.1 cm), ensuring that the ovary remained in its original position during measurement.

### Microscopic–anatomical examination

The assessment involved stretching organ samples (*n* = 34) with 14G needles, affixing them to a Styrofoam board, and immersing them in formalin (Lilly’s solution) at a 1:10 tissue-to-fixative ratio. Subsequently, the samples were cross-sectioned every 5 cm or in each of five macroscopic segments to obtain short oviducts. They were fully fixed in neutral-buffered formalin at 4 °C for 72 h and then moved to a pH-neutral phosphate buffer for further processing. Dehydration and paraffin embedding were performed using an embedding machine (Microm Laborgeräte GmbH, Heidelberg), and the samples were cured and stored at 4 °C for at least 12 h. Blocks were placed on a cooling plate at − 7 °C using a microtome (Microtome, Reichardt Jung AG, Heidelberg), before cutting 5-µm-thick sections were prepared. The sections were then stretched in a 38 °C water bath, transferred to slides, and coated with 3-aminopropyltriethoxysilane. The slides were dried and stored in sealed containers. The specimens were dried in an incubator at 45 °C for at least 24 h before hematoxylin–eosin staining (Memmert, Type: ST 40, V 220; Hz 50; W 2000). Finally, a hematoxylin–eosin stain was applied [22]. A Zeiss Axioskop 50 light microscope in conjunction with a Sony CCD-IRIS video camera was used for histological examination. The captured images were transmitted to a DELL Optiplex GX240 computer. The images were saved at a resolution of 768 × 574 dpi in 24-bit RGB format and subsequently analyzed using the Analysis 2.1 software. The type of epithelium present in the oviduct sections was also determined. The epithelial area was measured in three sections per location at a magnification of 20×, and the average height was calculated. The maximum and minimum heights were recorded to assess the height variation. The number of primary folds per 1000 μm was counted at either 2.5× or 5× magnification.

### Statistical analysis

The data collected in this study were meticulously recorded in Excel spreadsheets. Subsequently, statistical analyses were conducted using SPSS (version 15.0; SPSS Software GmbH, Munich, Germany). Initially, the Shapiro–Wilk Test was employed to determine if the data followed a normal distribution, specifically applied to serum estradiol-17ß and serum progesterone concentrations, as well as various oviduct measurements. For normally distributed data, ANOVA with Bonferroni Correction was applied to compare means among multiple groups, primarily concentrating on various oviduct measurements. To compare differences between two independent groups for serum hormone concentrations during the reproductive cycle, the Mann–Whitney U Test was utilized. Finally, Pearson’s Correlation was used to measure the linear correlation between different analyzed parameters, such as serum hormone concentrations and anatomical measurements of the oviduct. Throughout this study, all data are presented as means ± standard deviation (SD). Differences were considered statistically significant at *p* < 0.05.

## Results

### Estradiol-17ß and progesterone concentrations

**Fig. 1:**
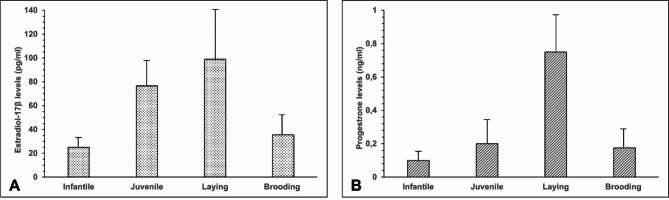
Arithmetic means and standard deviations of estradiol-17ß (pg/ml) and progesterone (ng/ml) concentrations in the blood serum of female Muscovy ducks during their reproductive periods. Significant differences were observed between infantile and juvenile ducks, and laying ducks (*p* = 0.001), as well as between laying and brooding ducks (*p* = 0.001) for Estradiol-17ß (A). Similarly, progesterone levels significantly differed between infantile and juvenile ducks, and laying ducks, and also between laying and brooding ducks (*p* < 0.001) (B). (*n* = 35)

Estradiol-17ß levels showed significant differences, with a mean of 76.7 ± 23.7 pg/ml (SD) in juvenile Muscovy ducks and a mean of 25.1 ± 6.9 pg/ml (SD) in infantile Muscovy ducks. This difference was statistically significant (*p* = 0.043). Additionally, comparison of serum estradiol-17ß concentrations across reproductive periods revealed significant differences. Ducks before sexual maturity had an estradiol-17ß concentration of 41.2 pg/ml. During the laying period, the concentration increased significantly to 138.0 pg/ml (an increase of 96.8 pg/ml, *p* = 0.001). In brooding ducks, the estradiol-17ß concentration decreased significantly to 58.4 pg/ml (a drop of 79.6 pg/ml, *p* = 0.001). Furthermore, the comparison of median serum estradiol-17ß concentrations across different reproductive stages revealed significant differences. Ducks prior to sexual maturity displayed a median estradiol-17ß concentration of 41.2 pg/ml. During the laying period, the median estradiol-17ß level increased to 138.0 pg/ml, representing a statistically significant rise of 96.8 pg/ml (*p* = 0.001). In brooding animals, a statistically significant decrease (*p* = 0.001) was observed, with the median estradiol-17ß concentration dropping to 58.4 pg/ml. This reduction of 79.6 pg/ml was also statistically significant (*p* = 0.001) (Fig. 1, A). Regarding progesterone, serum levels showed a significant increase (*p* = 0.046) throughout the reproductive cycle. Infantile ducks displayed a mean progesterone level of 0.1 ± 0.01 ng/ml (SD), which rose to a mean of 0.2 ± 0.05 ng/ml (SD) in juvenile ducks, peaking during the laying period. Ducks laying their first egg had a mean progesterone concentration of 0.9 ± 0.1 ng/ml (SD), which further increased to a mean of 1.3 ± 0.3 ng/ml (SD) after the sixth egg, before decreasing to a mean of 0.6 ± 0.3 ng/ml (SD) after the 20th egg, with no significant differences observed between these phases. During the brooding period, the progesterone concentration further decreased to a mean of 0.2 ± 0.1 ng/ml (SD). Throughout the remainder of the brooding phase, there was no significant (*p* = 1) change in the serum progesterone levels. Furthermore, significant differences were observed between the reproductive stages: 0.14 ng/ml before sexual maturity, 0.86 ng/ml during laying (*p* < 0.001), and a significant decrease to 0.1 ng/ml in the breeding period (*p* < 0.001). Additionally, The three phases of the reproductive cycle showed distinct differences in serum progesterone concentrations. Ducks that had not yet completed their sexual maturation exhibited a median progesterone level of 0.14 ng/ml. This value differed significantly from the median progesterone concentration observed in laying ducks, which was 0.86 ng/ml (*p* < 0.001). During the transition from the laying phase to the brooding phase, a significant decrease (*p* < 0.001) in the median progesterone level to 0.1 ng/ml was observed (Fig. 1, B).

### Macroscopic findings of the oviduct

**Fig. 2:**
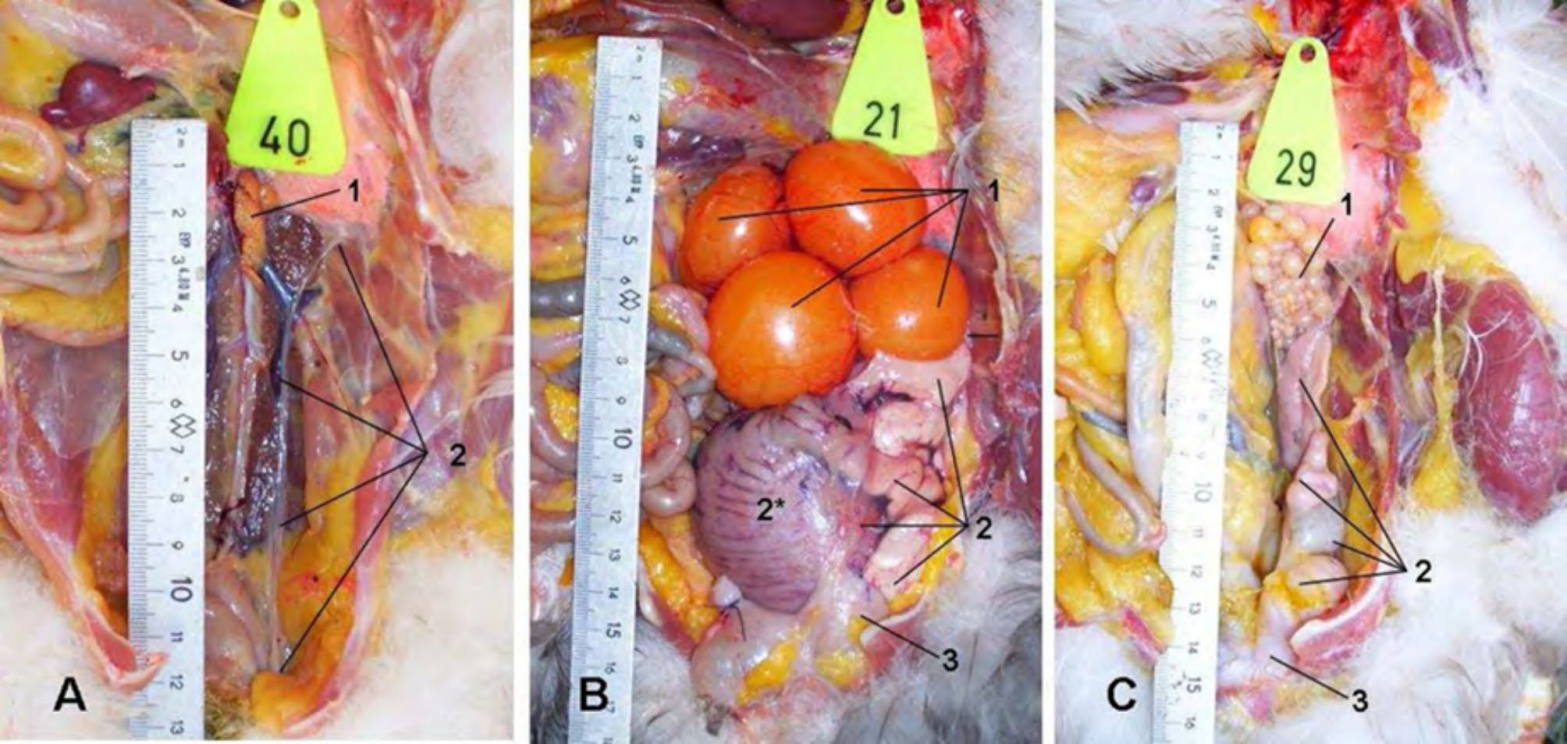
Oviducts of an infantile (**A**), laying (**B**), and 35-day brooding Muscovy duck in situ. The sternum and ventral portions of the abdominal air sac were removed. Visible structures include the left ovary (1), oviduct (2) with an egg still lacking a shell in the shell gland (2*), and the urodaeum (3) of the cloaca

In immature animals, the oviduct was observed as a thin fibrous tube on the dorsal wall of the coelomic cavity. In laying animals, it nearly filled the left caudal peritoneal cavity with extensive convoluted loops. In brooding animals, the oviduct appeared as a slightly twisted tube. The left oviduct was suspended in the coelomic cavity using the ventral and dorsal ligament oviducts (Fig. 2).

**Fig. 3:**
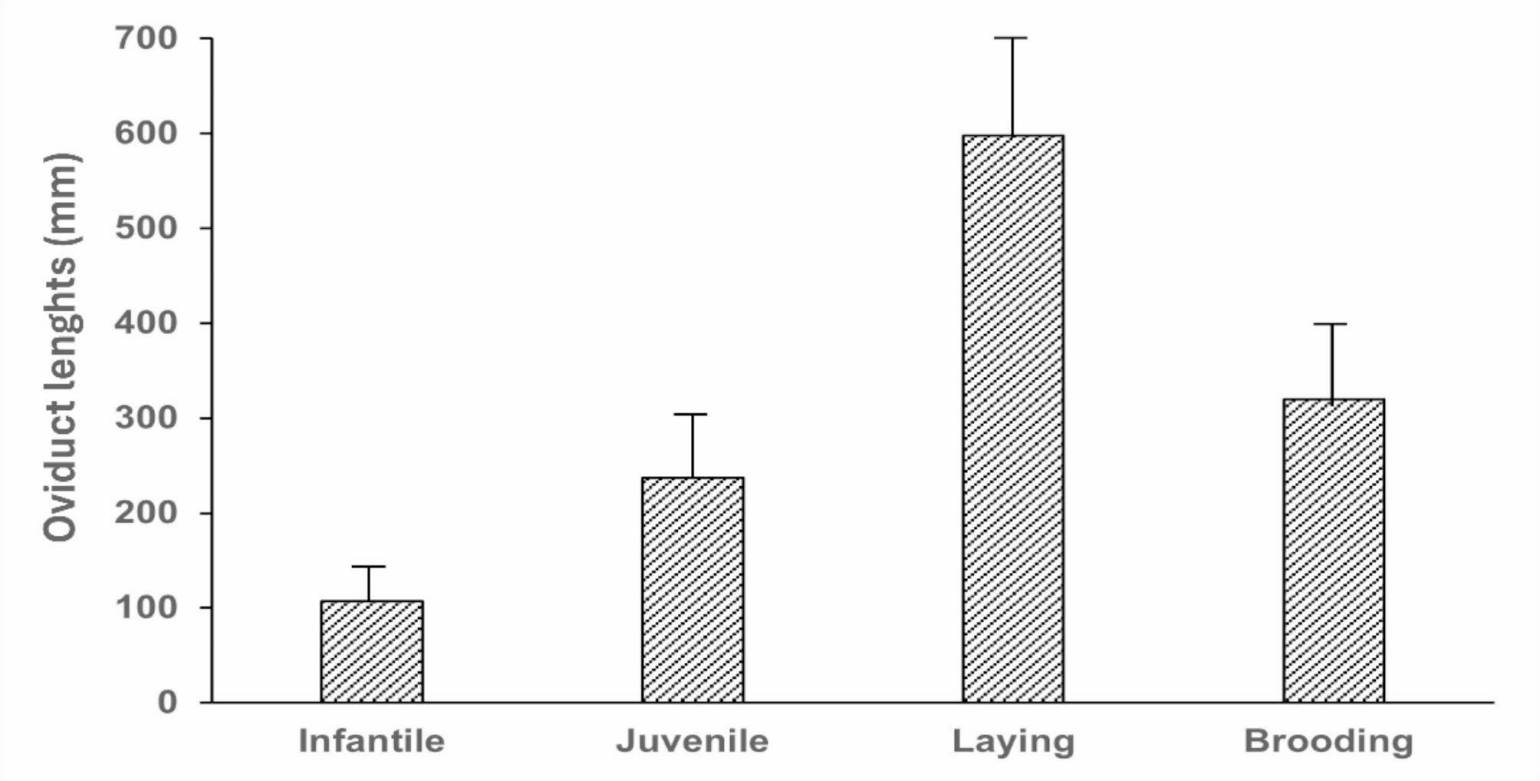
Arithmetic means and standard deviations of oviduct lengths in female Muscovy

Figure 3 illustrates the segmentation of the oviduct into distinct regions: the infundibulum, magnum, isthmus, shell gland, and vagina: The mean oviduct length in juvenile ducks was 237 mm (SD ± 122), which was significantly greater than the mean oviduct length in infantile ducks at 107 mm (SD ± 9, *p* = 0.002). Upon reaching sexual maturity, the mean oviduct length showed a further significant increase. Upon reaching sexual maturity, there was a significant increase (*p* = 0.002) in the mean oviduct length. The juvenile duck slaughtered at 275 days old had an oviduct length of 135 mm, the one slaughtered at 286 days old had an oviduct length of 205 mm, and the one at 289 days old had an oviduct length of 372 mm. Ducks that had laid their first egg exhibited a mean oviduct length of 566 ± 13 mm (SD). After laying the second egg, the mean oviduct length was 597 ± 37 mm (SD), and it decreased to 565 ± 52 mm (SD) after the fourth egg. During the egg-laying period, the mean oviduct length steadily increased to 632 ± 58 mm (SD) by the time the tenth egg was laid and then decreased to 567 ± 99 mm (SD) after the twentieth egg. The changes in mean oviduct length between the different phases of the egg-laying period were not significantly different (*p* = 1). However, following the fourth egg, the mean oviduct length progressively decreased to 565 ± 52 mm (SD) and continued to shrink until the end of the breeding season. After entering the brooding period, the mean oviduct length shortened to 404 ± 129 mm (SD). During the brooding period, the mean oviduct length further shortened. On the last day of brooding, the mean oviduct length averaged 235 ± 87 mm (SD), which was significantly shorter (*p* < 0.001) than those in laying ducks.

ducks relative to the reproductive stages (*n* = 35).

**Fig. 4:**
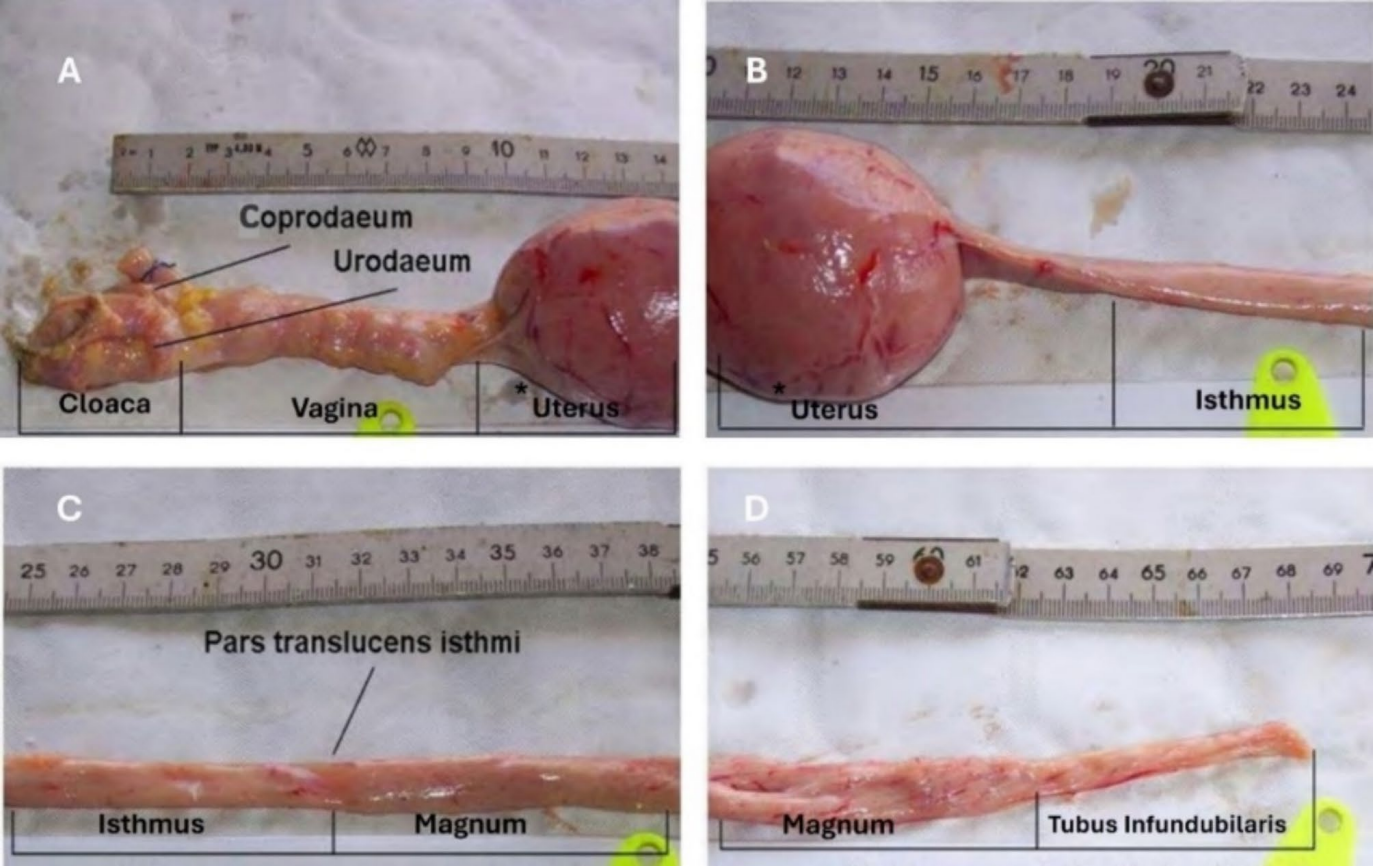
Transitions among individual oviduct segments (**A**-**D**) in a Muscovy duck, except the funnel-shaped enlargement of the infundibulum. *shell gland with egg

Figure 4 illustrates the following details: the tubular vaginal segment featuring rings (A) extends from the purple shell gland to cloaca, while the isthmus-to-shell gland region narrows and transitions from yellow pink to reddish-violet (B). The junction between the magnum and isthmus is characterized by connective tissue, with the isthmus exhibiting a yellowish-pink coloration and possessing finer spiral folds compared to the magnum (C). The magnum itself presents a pinkish-gray appearance with a reddish tint, a highly vascularized surface, and spiral folds that run along its entire length (D).

### Microscopic findings of the oviduct

**Fig. 5:**
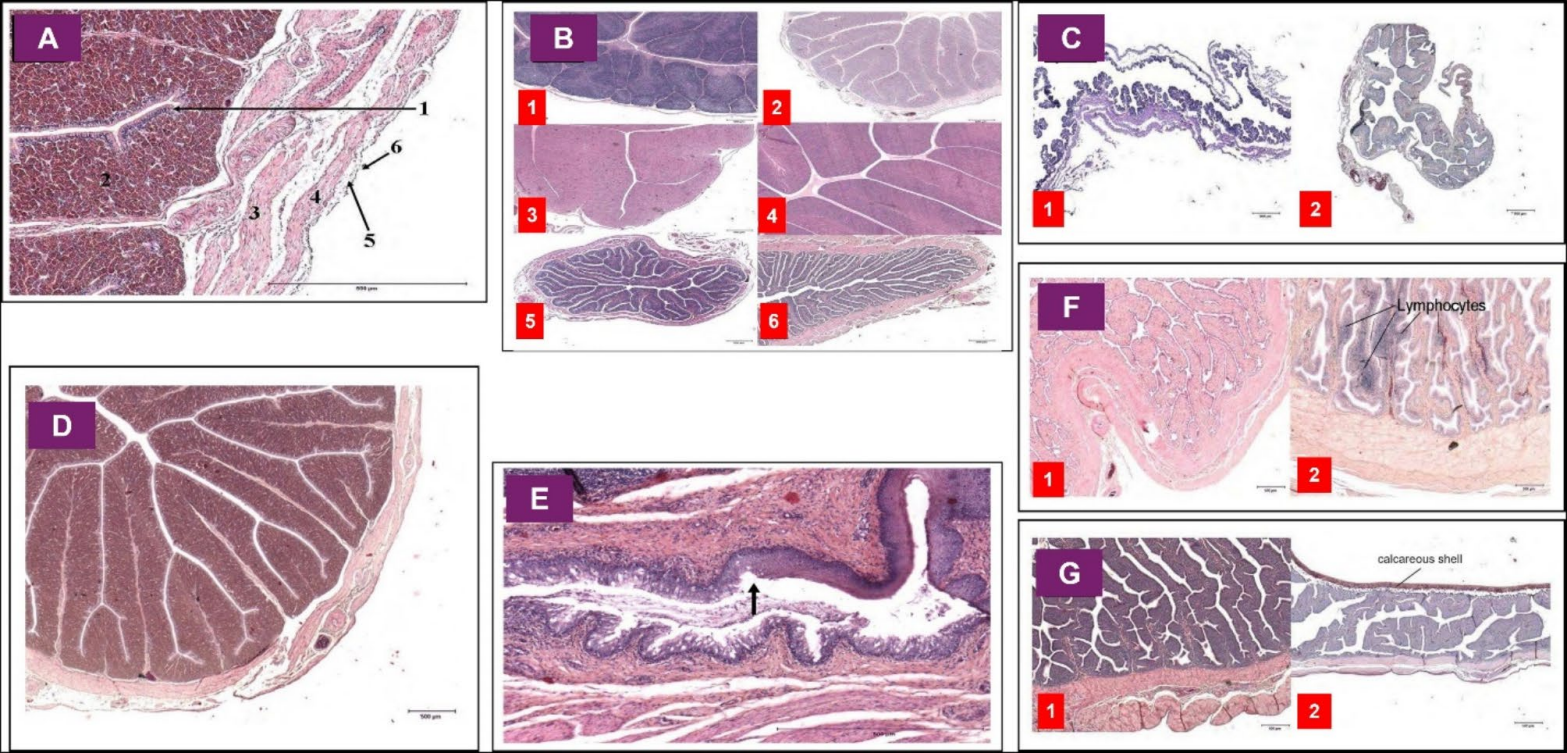
The histological cross-sections of Muscovy ducks are demonstrated with corresponding numbers for microscopic descriptions as follows: In section **A**, the isthmus in a laying Muscovy duck’s oviduct shows the tunica mucosa (lamina epithelialis mucosae (1) and lamina propria mucosae (2)), tunica muscularis (stratum circulare (3) and stratum longitudinale (4)), and tunica serosa (lamina propria serosae (5) and epithelium serosae (6)). Section **B** displays the magnum region in Muscovy ducks at various stages: infantile (1), juvenile (2), laying after the 2nd (3) and 20th egg (4), and brooding for 5 days (5) and 35 days (6). Section C presents the ovarian (1) and abovarian (2) infundibulum in a laying Muscovy duck. Section D illustrates the isthmus in a laying duck’s oviduct after laying the 6th egg. In section E, the urodeal region in a laying duck after the 15th egg is depicted, with an arrow marking the transition from oviduct epithelium (right) to cloacal epithelium (left). Section F shows the shell gland-proximal (1) and cloaca-proximal (2) vaginal regions in a laying duck after the 6th egg. Finally, section G illustrates the shell gland in a laying duck after the 6th egg (1) and the 10th egg (2), with part 2 showing the shell gland during egg passage, where the calcareous shell is visible; other egg components were removed during preparation. All sections are stained with **H**&**E**

To gather microscopic findings, 1.047 cross-sections from 349 locations in the oviducts of 35 Muscovy ducks were morphometrically analyzed. The analysis included 135 sections from the infundibulum, 435 from the magnum, 147 from the isthmus, 186 from the uterus, and 144 from the vagina. The findings from these histological cross-sections, along with their corresponding numerical identifiers for microscopic descriptions, are illustrated in Fig. 5.

**Fig. 6:**
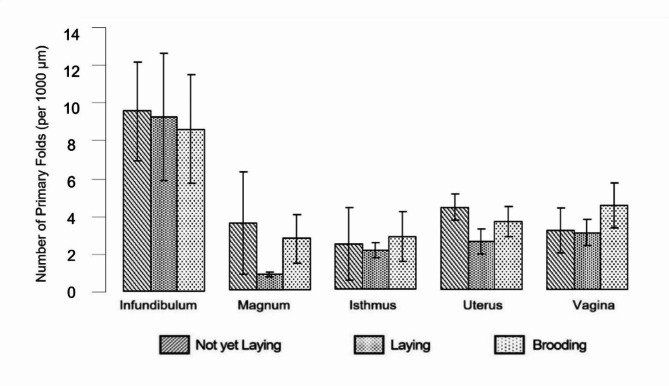
Primary fold numbers per 1000 μm of the individual oviduct sections dependent on the reproductive stages of Muscovy ducks, (*n* = 35)

The average number of primary folds per 1000 μm in different oviduct sections of female Muscovy ducks varied across reproductive cycles and significantly decreased in the magnum and shell gland during the laying period. In the infundibulum, the mean fold numbers differed significantly between infantile and juvenile ducks as non-laying birds (mean: 9.23, SD: ±2.55) and laying ducks (mean: 8.90, SD: ±3.27), with a p-value of < 0.001. In the magnum, the mean numbers decreased significantly from non-laying ducks (3.39 ± 2.62, SD) to laying ducks (0.75 ± 0.13, SD) (*p* < 0.001) and increased again in brooding ducks (2.61 ± 1.25, SD) (*p* = 0.002). For the isthmus, significant differences were observed between laying ducks (1.98 ± 0.39, SD) and brooding ducks (2.68 ± 1.29, SD) (*p* = 0.004). In the uterus, the mean fold numbers were significantly different between non-laying ducks (4.20 ± 0.69, SD) and laying ducks (2.42 ± 0.65, SD) (*p* < 0.001), as well as between laying and brooding ducks (3.48 ± 0.77, SD) (*p* = 0.04). In the vagina, no significant differences in mean fold numbers were noted between reproductive cycles. Figure 6 illustrates these variations across different sections and reproductive stages in female Muscovy ducks.

**Fig. 7:**
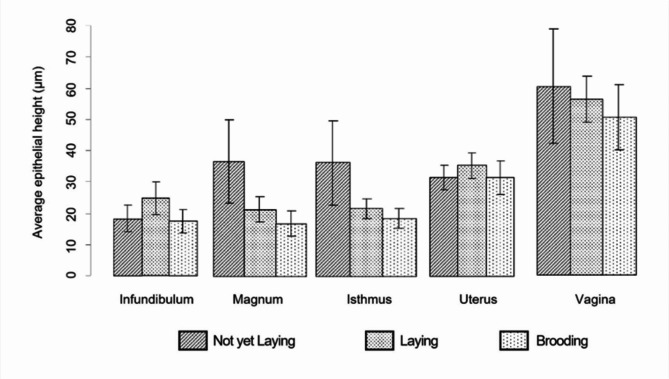
Average epithelial height (µm) of the individual cells of oviduct sections dependent on the reproductive stages of Muscovy ducks, (*n* = 35)

In addition, as detailed in Fig. 7, the mean epithelial heights (µm) and their standard deviations (SD) were assessed across the oviduct sections of female Muscovy ducks (*n* = 35) during different reproductive stages: non-laying, laying, and brooding. Significant differences were observed in some sections, highlighting the dynamic changes occurring throughout the reproductive cycle. In the infundibulum, the mean epithelial height was 15.78 μm (SD ± 3.73) during the non-laying stage, which increased to 21.33 μm (SD ± 4.44) in the laying stage. During the brooding stage, the value decreased again to 15.16 μm (SD ± 3.17). Significant differences were identified between the laying and brooding stages (*p* = 0.006). In the magnum, the epithelial height was highest during the non-laying stage with a mean of 33.58 μm (SD ± 12.24). This value significantly decreased to 19.67 μm (SD ± 3.80) during the laying stage (*p* < 0.001 compared to non-laying) and further declined to 15.52 μm (SD ± 3.61) during brooding. For the isthmus, the epithelial height followed a similar trend, measuring 33.25 μm (SD ± 12.35) during the non-laying stage, which significantly reduced to 19.71 μm (SD ± 2.87) during laying (*p* < 0.001 compared to non-laying) and to 16.94 μm (SD ± 2.96) in the brooding stage. In the uterus, the epithelial height remained relatively stable across stages. During the non-laying stage, it measured 28.25 μm (SD ± 3.57), increased slightly to 31.62 μm (SD ± 3.71) during the laying stage, and then decreased to 28.24 μm (SD ± 4.83) during brooding. No significant differences were noted for this section. Finally, in the vagina, epithelial height showed a progressive decrease across stages. It was highest during the non-laying stage at 57.41 μm (SD ± 17.34), reduced to 53.38 μm (SD ± 7.13) in the laying stage, and further decreased to 48.11 μm (SD ± 9.97) during brooding. However, these changes were not statistically significant.

**Fig. 8:**
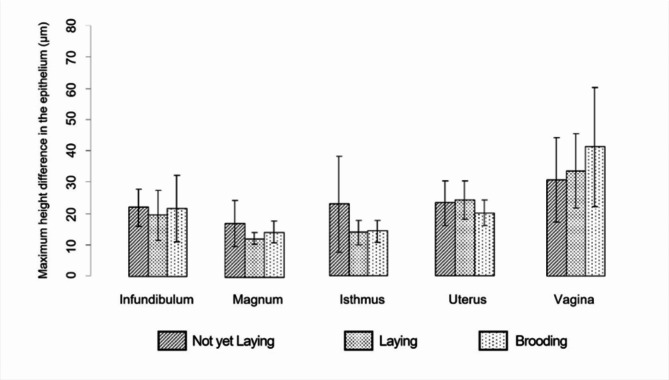
Maximum disparity in epithelial height (µm) among the various oviduct sections in relation to reproductive stages of Muscovy ducks, (*n* = 35)

As illustrated in Fig. 8, the maximal differences in epithelial height (µm) varied across the oviduct sections of female Muscovy ducks (*n* = 35) during their reproductive cycle stages: non-laying, laying, and brooding. In the infundibulum, the mean epithelial height during the non-laying stage was 15.76 μm (SD ± 4.18), which decreased slightly to 14.01 μm (SD ± 5.84) during the laying stage and returned to a comparable level of 15.64 μm (SD ± 7.72) in the brooding stage. Despite these changes, no significant differences were noted for this section. Meanwhile, in the magnum, the epithelial height showed a significant reduction as ducks transitioned from the non-laying stage (mean 12.24 μm, SD ± 5.31) to the laying stage (mean 8.90 μm, SD ± 1.34, *p* = 0.034), followed by a slight increase during the brooding stage (mean 10.43 μm, SD ± 2.45). For the isthmus, epithelial height was highest during the non-laying stage (mean 16.35 μm, SD ± 11.11), while it decreased significantly to 9.91 μm (SD ± 2.82, *p* = 0.046) during the laying stage, and remained stable at 10.17 μm (SD ± 2.59) during the brooding stage. In the uterus, the epithelial height fluctuated slightly across stages. It measured 16.73 μm (SD ± 5.15) in non-laying ducks, increased to 17.34 μm (SD ± 4.28) during laying, and decreased to 14.43 μm (SD ± 2.93) during brooding. However, these changes were not statistically significant. Finally, in the vagina, epithelial height increased steadily across the reproductive stages. It measured 22.10 μm (SD ± 9.67) during the non-laying stage, rose to 24.16 μm (SD ± 8.53) during laying, and reached its highest value at 29.58 μm (SD ± 13.67) during brooding. Despite the upward trend, no significant differences were observed for this section. Overall, the results highlight the dynamic variations in epithelial height across different oviduct sections, particularly in the magnum and isthmus, where significant changes were observed as ducks progressed through their reproductive cycle.

### Correlations between hormone concentration and macroscopic and microscopic findings

Estradiol-17β serum levels in Muscovy ducks showed significant positive correlations with oviduct length (*r* = 0.635; *p* < 0.001), average oviduct diameter (*r* = 0.798; *p* < 0.001), and the diameter of the dominant yolk sphere in the ovary (*r* = 0.741; *p* < 0.001). Additionally, there was a significant positive correlation with average epithelial thickness in the infundibulum (*r* = 0.554; *p* = 0.001). Conversely, estradiol-17β levels were negatively correlated with the number of primary folds per 1000 μm in the magnum (*r* = -0.655; *p* < 0.001) and the shell gland (*r* = -0.516; *p* = 0.003). Progesterone serum levels also correlated positively with oviduct length (*r* = 0.733; *p* < 0.001), average oviduct diameter (*r* = 0.832; *p* < 0.001), and the diameter of the dominant yolk sphere (*r* = 0.782; *p* < 0.001). There was also a positive correlation with average epithelial thickness in the infundibulum (*r* = 0.430; *p* = 0.014). However, progesterone levels were negatively correlated with the number of primary folds per 1000 μm in the magnum (*r* = -0.753; *p* < 0.001) and the shell gland (*r* = -0.559; *p* = 0.001). Overall, significant negative correlations were observed between the number of primary folds per 1000 μm in the oviduct and both oviduct length (*r* = -0.554; *p* = 0.001) and average oviduct diameter (*r* = -0.504; *p* = 0.002).

## Discussion

Hormonal fluctuations play a crucial role in the morphological changes observed in the female poultry reproductive system during the reproductive cycle. Given the increasing demand for poultry meat, it is important to understand these changes [15–17]. This study focused on assessing the reproductive capacity of female Muscovy ducks *(Cairina moschata forma domestica)*, also analyzing the concentrations of estradiol-17β and progesterone and examining the anatomical features of the oviduct. In general, the results of this study showed that hormone analysis revealed fluctuations in serum estradiol-17β and progesterone levels during the reproductive cycle, providing important insights for improving reproductive management and increasing productivity in Muscovy ducks. In this context, prior to the laying period, the levels of estradiol-17β in Muscovy duck blood serum are relatively low [23]. Infantile and juvenile Muscovy ducks at 70 days of age exhibit an average estradiol-17β concentration of 25.1 pg/ml. In contrast, the American black duck *(Anas rubripes)* shows significantly higher concentrations, averaging 70 pg/ml shortly after brooding [23]. As Muscovy ducks progress through sexual development, estradiol-17β levels increase, peaking during the laying period when the average afternoon concentration reaches 138.4 pg/ml. This pattern is consistent with that of the laying period in mallard-like ducks, where estradiol-17β concentrations fluctuate between 100 and 250 pg/ml throughout the day [24]. Notably, these observations differ from the serum estradiol-17β levels in red-breasted mergansers *(Mergus serrator)*, a type of diving duck. In addition, during the transition to the laying period in red-breasted mergansers, blood serum estradiol-17β concentrations decrease from 100 to 160 pg/ml to 40–60 pg/ml. In contrast, Muscovy ducks do not exhibit low concentrations until after the laying period [25]. In addition, to compare the progesterone levels during the brooding and egg-laying phases in other study studies, it was observed that during the brooding phase, progesterone levels were lower compared to ducks during their egg-laying phase [26]. Specifically, the progesterone levels were significantly reduced during the brooding phase, while they were higher during the egg-laying phase. In our research, the serum progesterone concentration increased steadily from infantile and juvenile ducks (0.1 ± 0.01 ng/ml) to adolescent (0.2 ± 0.05 ng/ml) ducks and continued to rise during the laying phase. During the laying period, ducks that laid their first egg had a progesterone level of 0.9 ± 0.1 ng/ml. After laying their 6th egg, the highest progesterone level was observed at 1.3 ± 0.3 ng/ml, which then decreased to 0.6 ± 0.3 ng/ml after laying their 20th egg. After entering the brooding period, the progesterone concentration further decreased to 0.2 ± 0.1 ng/ml. There were no significant changes in the serum progesterone levels throughout the brooding period. Thus, our results agree with these findings, showing that progesterone levels are higher during the egg-laying phase and decrease significantly during the brooding phase.

The oviduct wall in Muscovy ducks is structured into three layers: tunica mucosa, tunica muscularis, and tunica serosa, a composition that is consistent with that found in all other avian species [6, 27]. Similar to that in other avian species, the tunica mucosae can differentiate into lamina epithelialis mucosae and lamina propria mucosae [8, 28]. An assessment of the primary fold numbers in the segments of the avian oviduct, as conducted in the present study, has not been performed in other bird species. Furthermore, the epithelium is characterized by a single-layered iso- to columnar epithelium, which is also found in turkeys [5]. However, there are sections with multilayered epithelium. The average epithelial height in the infundibulum of infantile and juvenile Muscovy ducks is only half that of chickens [29] and mallards [30].

The magnum of Muscovy ducks has wide, blunt primary folds without secondary folding, unlike the narrow, secondary folded mucosal folds of domestic fowl [31, 32]. Similar fold changes have been observed in chickens [32, 33] and turkeys [27]. The magnum epithelium is single-layered, iso- to high-prismatic, similar to that in domestic fowls [32], but different from the multilayered epithelium in turkeys, guinea fowl, and pigeons [5, 27]. The magnal epithelium of infantile and juvenile Muscovy ducks before the onset of laying is 24% thicker than that of mallard ducks during the same cycle period. During laying, it flattens to 19.67 ± 3.83 μm, 47% of the height in laying female mallards [3] and similar to that in laying chickens [12]. The epithelium of waterfowl, including Muscovy ducks and female mallards, contains alternating goblet and ciliated cells [34], whereas chickens and pigeons mostly contain ciliated cells [33]. The lamina propria is similar in chickens [34], female mallards [6, 35], guinea fowl [27], and Muscovy ducks. However, ducts, like those found in guinea fowl [27], are not present in Muscovy ducks. Regarding the mucosal folds in the isthmus of Muscovy ducks, the results are similar to those in chickens, but lower, pointed, and narrower than those in the magnum. Unlike those in chickens, the primary isthmus folds in Muscovy ducks lack incisions [32] and secondary folds [31]. Furthermore, the epithelium of the isthmus in Muscovy ducks, as in all poultry species, is stratified columnar [29], which is 31% thicker in Muscovy ducks before the laying period begins compared to mallard ducks. During laying, it reduces to 74% of the mallard’s epithelial height [3], which is 18% lower than that in laying chickens [12]. Epithelium consists of basal cells and equal parts of goblet and ciliated cells, resembling that in chickens [36], and more so than other ducks [34] and that in mallards [6], indicating higher exocrine activity. Unlike that in chickens [36], there is no clear boundary between the lamina epithelialis and lamina propria in Muscovy ducks.

The uterine mucosal folds in Muscovy ducks are high and secondarily folded, similar to those in mallards [30], unlike the flat folds in ducks [37]. The uterine epithelium is multilayered and highly prismatic, similar to that of other poultry [30, 38]. The average heights of the uterine epithelia in infantile and juvenile Muscovy ducks and mallard ducks are nearly the same, with only a 16% height difference. During the laying period, the epithelia of Muscovy ducks are 30% higher than those of mallard ducks [3] and 21% higher than those of laying hens [12]. In chickens and mallard ducks, it consists of basal cells and apical ciliated cells [30, 39]. In Muscovy ducks, the lamina propria contains densely packed tubular glands [6] and is less developed compared to the tissue regions within the magnum or isthmus. Compared with mallards, Muscovy ducks possess slender, elongated, and intricately folded mucosal folds, as opposed to the flat folds observed during uterovaginal transition [40]. The vaginal epithelium of Muscovy ducks is distinguished by their multilayered and tall-prismatic composition, similar to that of chickens [41] and is notably the tallest among that of Muscovy ducks and mallards. The height difference between infantile and juvenile ducks and laying birds is minimal [3], and the vaginal epithelium in chickens is thinner than that in other oviduct sections and only 40% of the epithelial height in laying Muscovy ducks [12]. Furthermore, its cellular composition is similar to that of chickens, turkeys [41], geese [42], and ducks [39], and the vagina has a gland-free lamina propria with sperm storage tubules [8, 41, 43].

Finally, physiological changes in the female genital tract during the reproductive cycle in domestic poultry species are morphologically correlated with hormonal status [23, 44–46]. Although hormonal manipulation of reproduction in poultry is not a common practice, it is conceivable and may become necessary, given the rapidly growing demand for poultry meat [16, 18]. Knowledge of physiological processes and values is essential to enable such interventions. As the reproductive hormone status of female ducks has not yet been investigated during their reproductive cycle, this study aimed to determine the levels of estradiol-17β and progesterone during this period and identify morphological correlates in the oviduct. Yolk follicles within the ovaries of female poultry are hormonally active, orchestrating a sequence of intricate processes. Theca cells play a pivotal role as they predominantly synthesize androgens, such as androstenedione. These androgens are subsequently conveyed to granulosa cells, where the enzyme aromatase converts them into estradiol-17β. Notably, granulosa cells also produce progesterone, but this function is activated only post-ovulation. During this phase, granulosa cells undergo a transformation, becoming granulosa lutein cells within the corpus luteum to support progesterone synthesis [47–49]. As the follicles grow, the synthesis of these sex hormones increases [23]. In the ovaries of infantile and juvenile Muscovy ducks, the enlargement of the dominant yolk follicle is positively correlated with the serum concentrations of estradiol-17β and serum progesterone concentration, which indicates that the endocrine functions of yolk follicles in Muscovy ducks are similar to those in chickens.

In conclusion, hormonal analysis revealed a marked increase in serum estradiol-17β and progesterone during the egg-laying phase, with a subsequent decrease in the brooding phase. Macroscopic evaluations showed significant changes in the oviduct’s length, peaking after the 10th egg was laid, and these changes correlated with hormone levels. Microscopic analysis provided further structural insights into the oviduct throughout the reproductive cycle. This study offers critical data to enhance reproductive management practices for Muscovy ducks. In summary, the findings provide a foundation for improving Muscovy duck productivity and pursuing biotechnological methods to modify reproductive processes. Future research may focus on biotechnological advancements to enhance reproductive efficiency and farm productivity.

## Data Availability

The data used in this study belongs to the authors and can be requested by contacting the corresponding author: Abbas.farshad@vetmed.uni-giessen.de.
